# I’m the Only Son She Got Left: Male Caregivers’ Experiences Managing Older Adults’ Post-Acute Care

**DOI:** 10.1093/geroni/igab046.347

**Published:** 2021-12-17

**Authors:** Michael Bueno, David Russell, Jo-Ana Chase

**Affiliations:** 1 University of Missouri - Columbia / University of California, Irvine, Placentia, California, United States; 2 Appalachian State University, Boone, North Carolina, United States; 3 University of Missouri, Columbia, Missouri, United States

## Abstract

Family caregivers (FCGs) play an integral role supporting older adults transitioning to post-acute care following a hospitalization. FCGs function as advocates, information agents, and most importantly, care managers and providers. Although, caregiving has been traditionally seen as a female role, men are increasingly undertaking these roles and responsibilities. This research addresses a gap in the existing literature by exploring the subjective experiences of male FCGs of older adults in the post-acute setting. Using data from two parent qualitative studies on caregiving in the post-acute setting (N=40), we conducted a qualitative secondary analysis using conventional content analysis of male caregiver participants’ interview data (n=11). Interviews explored the subjective experiences of male caregivers’ interactions with home health care supportive personnel and conducting medical/nursing tasks for older adults. Five themes emerged: areas of abandonment, financial needs, masculinity, organization of care, and preparation. These themes highlighted areas of both confidence and struggle for male FCGs and captured their unique experiences managing the care of an older adult in the post-acute setting. Furthermore, the themes illustrate male FCGs’ feelings of guilt, financial impact, work disruptions, and the perceived effect of masculinity on their caregiving role. Findings can inform clinicians’ provision of focused and tailored resources to meet the specific needs of male FCGs. Future research should explore the evolving experiences of male FCGs over time, particularly those FCGs of older adults with chronic illnesses.

